# Prediction of hydraulic conductivity loss from relative water loss: new insights into water storage of tree stems and branches

**DOI:** 10.1111/ppl.12790

**Published:** 2018-09-04

**Authors:** Sabine Rosner, Berthold Heinze, Tadeja Savi, Guillermina Dalla‐Salda

**Affiliations:** ^1^ Institute of Botany BOKU University Vienna Gregor Mendel Straße 33, 1180 Vienna Austria; ^2^ Department of Forest Genetics, Federal Research and Training Centre for Forests Natural Hazards and Landscape Seckendorff-Gudent-Weg 8, 1130 Vienna Austria; ^3^ Division of Viticulture and Pomology BOKU University Vienna Konrad Lorenz‐Straβe 24 3430 Tulln an der Donau Austria; ^4^ INTA, EEA Bariloche Grupo de Ecología Forestal San Carlos de Bariloche Río Negro Argentina

## Abstract

More frequently occurring, drought waves call for a deeper understanding of tree hydraulics and fast and easily applicable methods to measure drought stress. The aim of this study was to establish empirical relationships between the percent loss of hydraulic conductivity (PLC) and the relative water loss (RWL) in woody stem axes with different *P*_50_, i.e. the water potential (Ψ) that causes 50% conductivity loss. Branches and saplings of temperate conifer (*Picea abies*, *Larix decidua*) and angiosperm species (*Acer campestre*, *Fagus sylvatica* *, Populus* x *canescens*, *Populus tremula*, *Sorbus torminalis*) and trunk wood of mature *P. abies* trees were analyzed. *P*_50_ was calculated from hydraulic measurements following bench top dehydration or air injection. RWL and PLC were fitted by linear, quadratic or cubic equations. Species‐ or age‐specific RWLs at *P*_50_ varied between 10 and 25% and *P*_88_, the Ψ that causes 88% conductivity loss, between 18 and 44%. *P*_50_ was predicted from the relationship between Ψ and the RWL. The predictive quality for *P*_50_ across species was almost 1:1 (r^2^ = 0.99). The approach presented allows thus reliable and fast prediction of PLC from RWL. Branches and saplings with high hydraulic vulnerability tended to have lower RWLs at *P*_50_ and at *P*_88_. The results are discussed with regard to the different water storage capacities in sapwood and survival strategies under drought stress. Potential applications are screening trees for drought sensitivity and a fast interpretation of diurnal, seasonal or drought induced changes in xylem water content upon their impact on conductivity loss.

**Abbreviations**
BD: basic stem density
*K*s: sapwood area specific hydraulic conductivity
*P*_50_: water potential or application of positive pressure resulting in 50% conductivity loss
*P*_88_: water potential or application of positive pressure resulting in 88% conductivity loss
PLC: percent loss of conductivity
RWL: relative water loss
RWL at *P*_50_: relative water loss at the water potential when 50% conductivity is lost
RWL at *P*_88_: relative water loss at the water potential when 88% conductivity is lost
SW: weight at full saturation
SWC: saturated water content
Ψ: water potential or the negative of positive pressure applied in a pressure collar

## Introduction

The *P*_50_, defined as the water potential in the (secondary) xylem conduits resulting in 50% loss of hydraulic conductivity, is an important plant physiological parameter that can be used to assess hydraulic safety margins of tree species (Delzon and Cochard 2014). For many species, knowledge exists about the *P*_50_ and the shape of vulnerability curves, i.e. the percent loss of conductivity (PLC) plotted against the water potential (Ψ; Choat et al. 2012), but little is known about the corresponding relative water loss (RWL) in the woody plant parts. Woody parts in plants are important sites for water storage (Holbrook 1995). Water storage capacity, or capacitance, is the amount of water withdrawn from a tissue induced by a given decline in Ψ (Tyree and Yang 1990, Domec et al. 2006). Concerning plant survival, the capacitance aspect is of high relevance (McCulloh et al. 2014) because many species, especially angiosperms, operate at water potentials below *P*_50_ (Choat et al. 2012). These plant species may take advantage either of the capacitive water released from stored water in living wood tissues (Salomón et al. 2017) or from cavitation (Hölttä et al. 2009, Vergeynst et al. 2015). However, the latter process results in loss of hydraulic conductivity, which can be repaired by refilling when enough water is again provided from soil after rain events (Salomón et al. 2017). If the recovery from cavitation is possible under negative pressure is under debate (Brodersen et al. 2010, Cochard et al. 2013, Trifilò et al. 2015, Charrier et al. 2016, Brodersen et al. 2018). In order to better understand the role and functioning of cavitation, Vergeynst et al. (2015) underlined the need for datasets on capacitive water loss of different species at a wide range of water potentials; such datasets can be used to feed functional plant models (e.g. Huang et al. 2017, Salomón et al. 2017). Species‐specific resilience to drought can be only fully understood if models cover both ‘hydraulic proxies’ (safety margins) as well as ‘metabolic proxies’ that include the capacitance aspect and its relationship with embolism repair (Trifilò et al. 2015).

Capacitance is either expressed as the increase in the mass of water lost relative to the sample volume per unit Ψ change or as the increase in RWL per unit Ψ change, where the latter allows comparisons of samples differing in total water volume and wood density (Domec and Gartner 2001). Continuously drought stressed sapwood faces three phases: (1) water release from open conduits, damaged and non‐functional conduits (fatigued conduits) and intercellular spaces, (2) cavitation and release from elastic storage, and (3) the loss of tightly bound water after the wood has lost all of its hydraulic conductivity (Pratt and Jacobsen 2017). Capacitance of sapwood has been mainly calculated from experiments where small samples, often derived from coring, are allowed to dehydrate and where the Ψ is subsequently measured at decreasing water content by hygrometers (Trifilò et al. 2015, Savi et al. 2017) or thermocouple psychrometry (Meinzer et al. 2003, McCulloh et al. 2014, Jupa et al. 2016). Such curves obtained for sapwood have an exponential shape with extremely high‐water release at high Ψ. In contrast, water release curves of detached branches have rather a linear slope when plotted against Ψ (Domec and Gartner 2001, Gleason et al. 2014, Blackman et al. 2016). RWL curves assessed in parallel with PLC on exactly the same samples are available for trunk wood of *Pseudotsuga menziesii* (Domec and Gartner 2001, 2002) and *Picea abies* (Rosner et al. 2006). Furthermore, conductivity loss was successfully related to relative water loss in *Picea abies* branches (Hietz et al. 2008) but we still lack information for other conifer and angiosperm species. Recently, Pivovaroff et al. (2016) presented an automated approach to estimate the water release induced by centrifugation and found a strong relation between 50% water release (referred to the maximum possible water release induced by spinning) and conductivity loss in a conifer and short‐vessel species but not in a long‐vessel species.

The aim of this study was to establish empirical relationships between the PLC and the RWL in temperate conifer and angiosperm species differing in their vulnerability to cavitation. Measuring the PLC directly is challenging because hydraulic methods are very laborious, time consuming and bear potential risks of measurement errors due to, e.g. clogging of tracheids or vessels, or are restricted to specific facilities (Cochard et al. 2013). Although classical non‐automated point measurement techniques, such as bench top dehydration and air injection, or the recently developed pneumatic method (Zhang et al. 2018) are quite time consuming, they bear the opportunity to assess the water loss gravimetrically on the same specimen where the conductivity loss is measured. In order to test the predictive quality of RWL for PLC, the *P*_50_ calculated from direct flow measurements was compared to the empirically modeled *P*_50_ values. By establishing empirical relationships between RWL and PLC we were particularly aiming to get a fast prediction of PLC from RWL and extending our knowledge on (1) the amount of water that is stored in woody stems and branches, (2) the amount of water released at *P*_50_ and (3) the amount of water that is left over at *P*_88_ (i.e. Ψ at 88% conductivity loss) in species varying in vulnerability to cavitation and wood density. In this first approach, we screened two conifer and five angiosperm species. Different age classes of trunk wood and branches were explored in one conifer species.

## Material and methods

### Plant material

Plants came from the botanical garden at BOKU University (Vienna), from a site near the river ‘Wien’ in Vienna, and from southern Norway (Appendix S1). Branches and saplings were consecutively harvested during the summer seasons (June–August) from 2008 to 2017. All plants were well irrigated during the weeks before they were harvested. Beech saplings in BOKU botanical garden were irrigated throughout the whole growing period which can result in higher hydraulic vulnerabilities than reported from literature (Awad et al. 2010, Herbette et al. 2010). The dataset for the Norway spruce trunk wood samples came from an earlier study (Rosner et al. 2016). In total, seven different temperate species were investigated. Conifer species comprised *Picea abies* (L. Karst.) and *Larix decidua* Mill. In *P. abies*, small four‐year‐old trees as well as branches and trunk wood from the living crown of older trees were studied. Angiosperm species comprised: *Acer campestre* L., *Fagus sylvatica* L., *Populus* x *canescens* (Aiton) Sm., *Populus tremula* L., *Sorbus torminalis* (L.) Crantz. *Populus* x *canescens* is a natural hybrid between *P. alba* and *P. tremula*. Angiosperm species analyzed in this study have all a diffuse porous wood structure; however, *F. sylvatica* can develop a semi‐ring porosity in dependence of the growth situation. In Appendix S1, detailed description on the origin and age of the plant material is given. Selection criterion for angiosperm species was the access in botanical gardens near BOKU University, which guaranteed freshness of the material. We chose native species with a wide range in *P*_50_ (Choat et al. 2012, Rosner 2013) but avoided ring‐porous species because we expected artifacts with the air injection method regarding cut open vessels (Cochard et al. 2013).

### Hydraulic testing: air injection method

We harvested the whole above ground plant material in saplings and whole side branches were cut from their bigger supporting branches. Saplings or branches with a length varying from 0.5 to 1.5 m were harvested in the morning and transported to the laboratory in black plastic bags containing wet towels. In most cases, transport time took only some minutes and always less than 1 hour. Samples were sequentially re‐cut at both ends with scissors. Internodes with a length from 200 to 300 mm were cut and debarked under water and thereafter re‐saturated under low vacuum for 24 h at 4°C in filtered, distilled water (Hietz et al. 2008) containing 0.005% Micropur (Katadyn Products, Wallisellen, Switzerland) which makes use of silver ions to prevent microbial growth (Mayr et al. 2007). This method is appropriate to refill conduits in the functioning sapwood (Rosner et al. 2018). Prior to hydraulic conductivity measurements, specimens were re‐cut several times with a razor blade. Sapwood area specific hydraulic conductivity (*K*s, cm^2^ s^−1^ MPa^−1^) was measured under a hydraulic pressure head of 5.4 kPa with distilled and filtered (0.22 μm) water containing 0.005% Micropur. After the first conductivity measurement, the saturated weight (SW) of the specimen was determined on a balance (resolution of 0.0001 g, Mettler‐Toledo International Inc., Greifensee, Switzerland). Thereafter, air overpressure was applied to the specimens in a double‐ended pressure chamber (PMS Instruments, Corvallis, OR), the specimen was weighed again in order to determine the fresh weight (FW) at a given pressure application. After equilibration in distilled water for half an hour (Rosner et al. 2006), the hydraulic conductivity was measured again. The short exposition in distilled water leads to no increase in hydraulic conductivity as tested in pre‐trials. Initially, the pressure chamber was pressurized ranging from values of 0.5–2.0 MPa (in dependence of the hydraulic sensitivity of the species), and the pressure was increased after each conductivity measurement in steps of 0.5 or 1.0 MPa until more than 80% loss of conductivity was reached. The pressure exposition was standardized to 1 min; this exposure time was sufficient to result in the water loss according to a given pressure application. FW was assessed after each pressure application.

Hydraulic vulnerability curves of isolated conifer trunk wood samples were obtained with the methods described by Spicer and Gartner (1998) and Domec and Gartner (2001). We used a part of an existing dataset from an earlier study (Rosner et al. 2016). This part comprised a hydraulic vulnerability curve dataset of six healthy mature Norway spruce trees. Wood samples came from sapwood of the living crown and contained 2–3 complete annual rings. Tangential and radial faces of 200 mm long wood beams were shaved on a sliding microtome, thereafter shortened on a band saw and re‐cut several times with a razor blade. Specimens were kept wet during all preparation steps before they were re‐soaked as described above (Hietz et al. 2008). The final dimensions of the specimens were 6 (radial) × 6 (tangential) × 120 mm (longitudinal). Details for performing the measurements on standard size trunk wood samples can be found in detail in Rosner et al. (2006, 2016). The procedures to construct vulnerability curves and relative water loss curves are described below.

### Hydraulic testing: bench top dehydration method

In order to test the methodological impact (air injection vs. dehydration) on the relationship between RWL and PLC, hydraulic measurements were also performed after bench top dehydration with samples of *A. campestre*, *F. sylvatica, and P. tremula*. This classical method to construct vulnerability curves was introduced by Sperry et al. (1988) and is regarded as a reliable method for VCs measurements (Cochard et al. 2013, Torres‐Ruiz et al. 2014) in species where no clogging of vessels due to, e.g. the development of gels occurs (Jacobsen and Pratt 2012). Whole saplings and branches with a length of about 1 m were cut in the morning and transported to the laboratory as described above. They were rehydrated for 24 h in a dark room at 4°C by immersing their cut ends in fresh water, all other parts were covered with a black plastic bag. After re‐saturation, all water was removed from leaves and bark with paper towels, the cut end was sealed with Parafilm M^®^ (Bermis Company, Inc., Oshkosh, WI) and the branch or shoot was left dehydrating at ambient temperatures (24–26°C) for different time intervals in order to reach different Ψ and the corresponding conductivity losses. Ψ was estimated with a Scholander type pressure chamber (Plant Water Status Console from Soilmoisture Equipment Corp., Santa Barbara, CA) on leaves covered with aluminum foil for 1 hour prior to measurement. After half an hour of equilibration in a black plastic bag containing wet towels, the branch was progressively shortened, and 2–3 stem samples with a length of 5 cm were cut under water. Care was taken that the location of sampling was in a distance of at least 400 mm from the sealed cut end (Torres‐Ruiz et al. 2015). After de‐barking and re‐cutting the samples with a razor blade, the sapwood area specific hydraulic conductivity was measured with a hydraulic pressure head of 5.4 kPa and the specimens were weighed in order to determine the FW corresponding to the stem Ψ. Specimens were re‐saturated as described above (Hietz et al. 2008) and hydraulic conductivity at full saturation and SW were assessed. The approaches to construct vulnerability curves and water loss curves are described below.

### Relative water loss, saturated water content and basic wood density

After hydraulic testing, all specimens were dried at 103°C for 24 h (Rosner 2017). The relative water loss (RWL) at Ψ was calculated as:

(1) $$\mathrm{RWL}\;\left(\%\right)=100*\;\left(1–\left(\left(\mathrm{FW}–\mathrm{DW}\right)/\left(\mathrm{SW}–\mathrm{DW}\right)\right)\right)$$

SW is the weight at full saturation, FW is the weight at a given Ψ and DW is the dry weight. RWL is defined as the loss of water related to the water content in the fully saturated state, thus, in order to calculate reliable RWL values, it is absolutely necessary to have the fully saturated state as a reference. In that regard, it was intended to refill some conduits in the secondary xylem that might not be filled with free water out in the field, which should be avoided for other applications (Pivovaroff et al. 2016). Correct determination of the SW is crucial for the approach presented in this study because relating FW to an in situ water content that may vary with stem age, season, site and other factors (Hacke et al. 2015) would likely obscure the relationship between RWL and PLC. An unstandardized approach would not allow correct projection of PLC when using empirical models.

The saturated water content (SWC) was calculated as:

(2) $$\mathrm{SWC}\;\left(\%\right)=\left(\mathrm{SW}-\mathrm{DW}\right)/\mathrm{DW}*\;100$$

The basic stem density (BD) was calculated as the dry mass divided by the volume in the fully saturated state (Hietz et al. 2008) measured with a caliper (resolution of 0.1 mm). Details of BD measurements are described in Rosner (2017).

### Sample numbers, data processing and statistical analyses

The amount of sampled trees, sample numbers and the number of measurements for each species can be found in Appendix S1. Data processing and statistical analyses were carried out with SPSS™ 21.0. Data were tested for normal distribution with the Kolmogorov–Smirnov test. One‐way anova and post hoc Scheffé test were used to check for differences in mean values of BD, SWC and *K*s at full saturation between species or age classes. Relationships between traits or differences in mean values were accepted as significant if the *P*‐value was <0.05.

**Figure 1:**
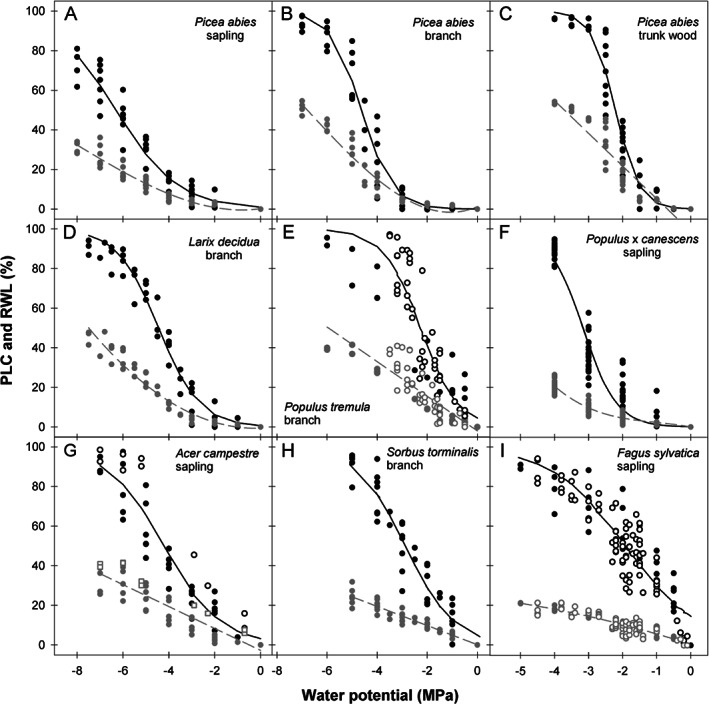
PLC (black symbols) and RWL curves (gray symbols) of *Picea abies* saplings (A), branches (B) and trunk wood (C), *Larix decidua* branches (D), *Populus tremula* branches (E), *Populus* x *canescens* saplings (F), *Acer campestre* saplings (G), *Sorbus torminalis* branches (H) and *Fagus sylvatica* saplings (I). Filled dots indicate datasets derived from the air injection method, open dots from the bench top dehydration method (E, G, I). Water potential refers thus either to the measurement with a Scholander type water status console or to the application of positive pressure in a double ended pressure collar. Please note the different scales in the *x*‐axis of the panels. Information on the regression lines can be found in Appendices S2 and S4.

Hydraulic conductivity at a given Ψ was related to the hydraulic conductivity at full saturation in order to calculate the percent loss of conductivity (PLC). The PLC pooled for each species or organ was plotted against the negative of the applied pressure or Ψ (Fig. 1). We found no evidence for cavitation fatigue (Hacke et al. 2001b) due to repeated pressure application because air injection PLC was not higher than bench top dehydration PLC for given pressure application or Ψ. Hydraulic vulnerability curves were fitted by an exponential sigmoidal equation (Pammenter and Vander Willigen 1998) to calculate *P*_50_ and *P*_88_, i.e. the pressure application at which 50 or 88% of conductivity loss occurred. The following equation was used:

(3) $$\mathrm{PLC}\;\left(\%\right)=100/\left(1+\exp\left(a*\left(\Psi -b\right)\right)\right)$$

The parameter ‘*a*’ corresponds to the slope of the linear part of the regression and ‘*b*’ is the *P*_50_. *P*_88_ can be calculated with these parameters. Ψ in Eqn. 3 is used here and thereafter for both water potential and the negative of the applied pressure in the pressure collar. Results for the slope and *P*_50_ are provided with their se and the 95% confidence interval (CI 95%; Appendix S2).

**Figure 2:**
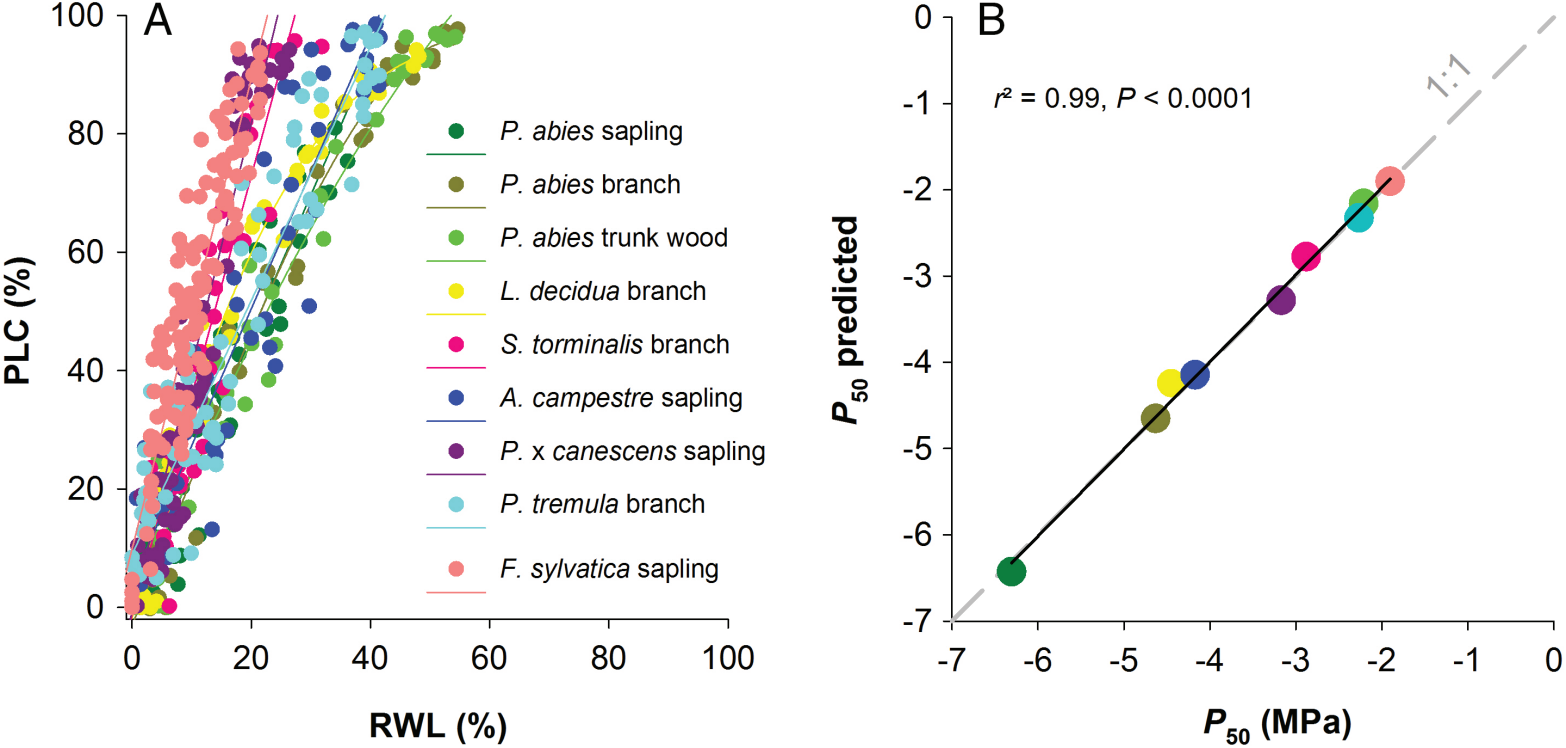
PLC plotted against the RWL for two conifer (*Picea abies*, *Larix decidua*) and five angiosperm species (*Acer campestre*, *Fagus sylvatica*, *Populus tremula*, *Populus* x *canescens*, *Sorbus torminalis*) (A) and the prediction of *P*_50_ from equations and parameters (RWL at *P*_50_) presented in Table 1 and Appendices S2 and S3 (B). Detailed information on the regression parameters for the scatter plots shown in (A) is given in Appendix S4.

The relationship between PLC and RWL (Fig. 2A) was tested by the ‘curve estimation’ function in SPSS™ 21.0. Linear, quadratic or cubic regressions were chosen according to their predictive quality. Quadratic equation was the best fitting for *L. decidua*, cubic fittings for *P. abies* branches and *P*. x *canescens* saplings. The equation calculated for each species or organ was used to calculate the RWL at *P*_50_. We provide the CI 95% and 95% individual prediction intervals (PI 95%) for RWL at *P*_50_ or *P*_88_ (Appendix S3).

Water loss curves, i.e. the RWL plotted against Ψ (RWL‐Ψ curve, Fig. 1) were fitted by the ‘curve estimation’ function in SPSS™ 21.0. We choose the fitting with the highest predictive quality (r^2^) and with a reliable shape. Curve shapes were linear, quadratic or cubic (Fig. 1). The ‘curve estimation’ function was also used to establish the relationship between Ψ and RWL in order to predict Ψ at a given RWL, for instance the RWL at *P*_50_ or at *P*_88_. In these curves, the Ψ was plotted against the RWL (Ψ‐RWL curve). Linear equations were applied in *P. abies* trunk wood and in branches of *A. campestre*, *P. tremula* and *S. torminalis*, quadratic equations in *F. sylvatica* saplings and branches of *L. decidua* and *P. abies*, and cubic equations in saplings of *P. abies* and *P*. × *canescens* (Appendix S4).

The predictive quality of the RWL for PLC across species or organs was tested by calculating an empirically modeled *P*_50_ from the RWL at *P*_50_ and the species‐, age‐ or organ‐specific relationship between Ψ and RWL (Ψ‐RWL curve). The same procedure was performed for *P*_88_. The predicted values of *P*_50_ and *P*_88_ are provided with their 95% CI and 95% PI (Appendix S4).

## Results

**Figure 3:**
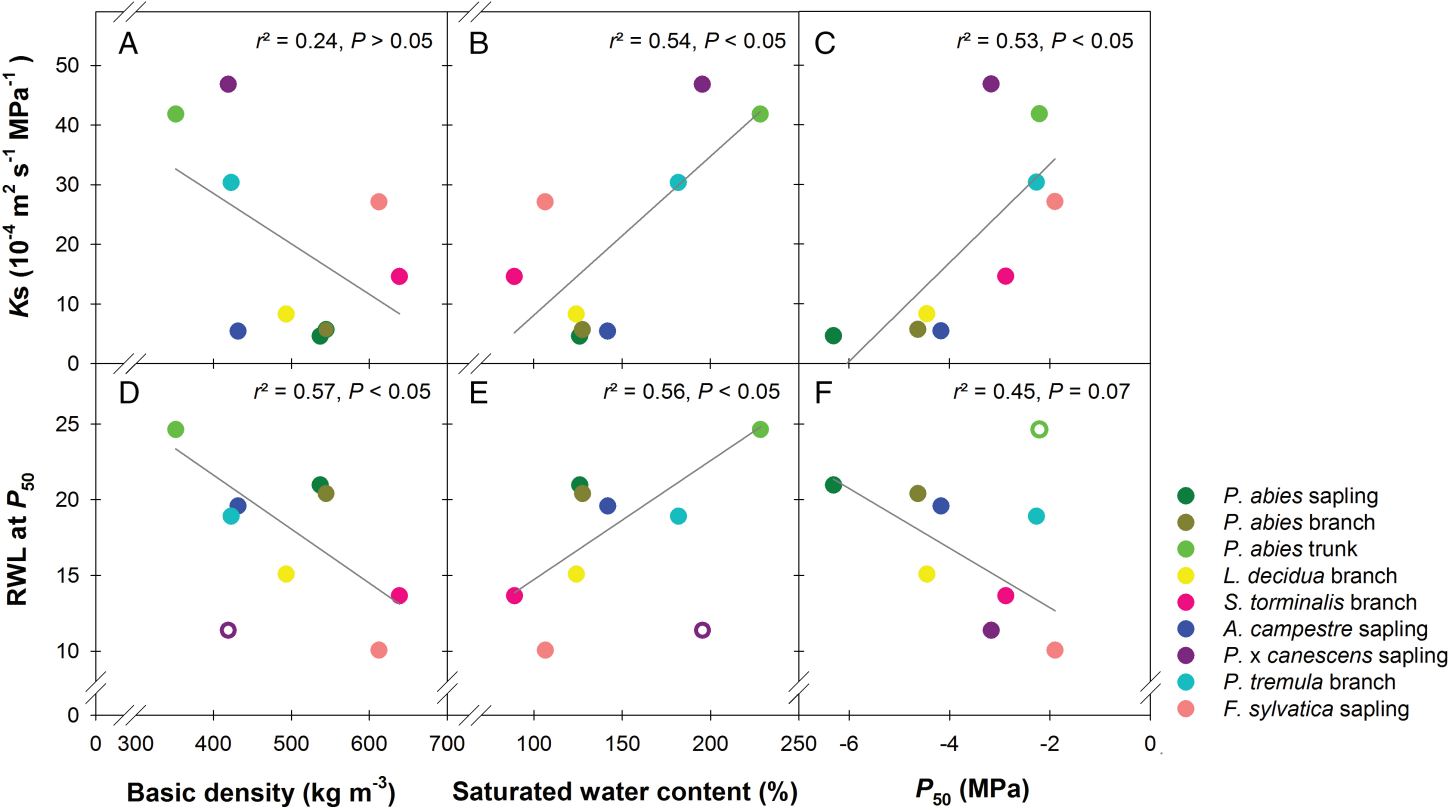
Relationships between the specific hydraulic conductivity at full saturation (*K*s) and basic density (A), the saturated water content (B) and *P*_50_ (C) and relationships between relative water loss at 50% percent loss of hydraulic conductivity (RWL at *P*_50_) and basic density (D), the saturated water content (E) and *P*_50_ (F) for two different conifer and five angiosperm species. The poplar hybrid *Populus* x *canescens* was excluded from regression analyses in plots (C) and (D), trunk wood of *Picea* *abies* in plot (F). Excluded species or age classes are indicated by open symbols.

Species examined in this study varied widely in their vulnerability to cavitation (Fig. 1, Table 1). The lowest *P*_50_ was calculated for young *P. abies* trees (−6.3 MPa) and *L. decidua*, the other studied conifer species, showed also quite low *P*_50_ in its branches (−4.4 MPa). The main trunk of older *P. abies* trees was however quite vulnerable with a *P*_50_ of −2.2 MPa. This *P*_50_ ranged around the values measured for *F. sylvatica* saplings (−1.9 MPa) and *P. tremula* branches (−2.3 MPa), which were the most vulnerable angiosperm species. Young *P*. x *canescens* trees and *S. torminalis* branches had *P*_50_ values around −3 MPa. The most resistant angiosperm species in this study was *A. campestre* (*P*_50_ = −4.2 MPa). The hydraulic measurements performed after bench top dehydration of the branches were well within the range of conductivity losses induced by air‐injection (Fig. 1E, G, I). In *F. sylvatica*, where enough point measurements were available to compare both methods (bench top dehydration and air injection), *P*_50_ varied only within a narrow range (from −1.9 to −1.8 MPa). BD varied across species and age classes and was strongly related to the SWC (r^2^ = 0.86, *P* < 0.001). SWC showed a significant relationship with *K*s (r^2^ = 0.54, *P* < 0.05, Fig. 3B). *P*_50_ was neither related to BD nor to SWC, because *F. sylvatica* and *S. torminalis* had highest BD but were also quite vulnerable (Table 1). *K*s was significantly positively related to *P*_50_ (r^2^ = 0.53, Fig. 3C) and *P*_88_ (r^2^ = 0.66, *P* < 0.05).

**Table 1:** Basic density (BD), saturated water content (SWC), sapwood area specific hydraulic conductivity at full saturation (*K*s), water potential/pressure application at 50% (*P*_50_) or 88% (*P*_88_) conductivity loss, and the RWL at 50% conductivity loss (RWL at *P*_50_) and 88% (RWL at *P*_88_) conductivity loss for seven different temperate woody species and different age classes. Mean values are given with their se. For BD, SWC and *K*s the post hoc Scheffé test was performed after one‐way anova (*P* < 0.0001). The same letters indicate no significant differences between species or age classes. Information on the statistics of the hydraulic vulnerability curves for predicting *P*_50_ and *P*_88_ are provided in Appendix S2. Equation parameters for calculation of RWL at *P*_50_ and RWL at *P*_88_ and their confidence‐ and predicting intervals are presented in Appendix S3.

Plant group/class	Species and organ	BD [kg m^−3^]	SWC [%]	*K*s [10^−4^ m^2^ s^−1^ MPa^−1^]	*P*_50_ [MPa]	*P*_88_ [MPa]	RWL at *P*_50_ [%]	RWL at *P*_88_ [%]
Conifers	*Picea abies* sapling	537.0 ± 19.6^d^	125.9 ± 6.8^bc^	4.6 ± 0.4^a^	−6.3	−9.0	21.0	36.2
	*Picea abies* branch	544.4 ± 6.6^de^	127.5 ± 3.7^bc^	5.7 ± 0.8^a^	−4.6	−5.8	20.4	43.6
	*Picea abies* trunk wood	351.4 ± 11.0^a^	228.3 ± 8.3^e^	41.8 ± 3.8^cd^	−2.2	−2.9	24.6	43.9
	*Larix decidua* branch	493.3 ± 11.6^cd^	124.0 ± 4.9^bc^	8.3 ± 0.9^a^	−4.4	−6.2	15.1	39.8
Angiosperms	*Acer campestre* sapling	431.5 ± 9.7^bc^	141.8 ± 4.8^c^	5.5 ± 1.8^a^	−4.2	−6.6	19.6	34.3
	*Fagus sylvatica* sapling	612.4 ± 5.0^ef^	106.4 ± 1.2^ab^	27.1 ± 0.9^bc^	−1.9	−4.1	10.1	18.2
	*Populus* x *canescens* sapling	418.3 ± 7.1^ab^	195.3 ± 2.2^d^	46.8 ± 2.8^d^	−3.2	−4.2	11.4	20.3
	*Populus tremula* branch	422.5 ± 11.8^abc^	181.9 ± 6.3^d^	30.4 ± 2.0^bcd^	−2.3	−3.7	18.9	34.9
	*Sorbus torminalis* branch	638.7 ± 15.6^f^	89.0 ± 2.2^a^	14.6 ± 1.0^ab^	−2.9	−4.8	13.7	23.0

The relationship between PLC and RWL was in general linear below about 80% PLC and was species‐ and age‐specific (Fig. 2A). Therefore, the RWL at *P*_50_ and *P*_88_ varied widely (Table 1). Lowest RWL at *P*_50_ values (10–11%) were found for *F. sylvatica* and *P*. x *canescens*, while slightly higher values were in *S. torminalis* and *L. decidua* (14–15%). *P. tremula*, *A. campestre* and *P. abies* saplings and branches had much higher RWL at *P*_50_ (19–21%). *P. abies* trunk wood (25%) had the highest RWL at *P*_50_ and no overlapping 95% confidence intervals with *P. abies* branches or saplings (Appendix S3). RWL at *P*_88_ varied from 18 to 44% (Table 1) and was positively related to RWL at *P*_50_ (r^2^ = 0.76, *P* < 0.01). In general, conifers had highest RWLs at *P*_88_. The relationship between PLC and RWL was neither influenced by the method to assess hydraulic vulnerability curves (Appendix S5) nor was it inconsistent across time, as proved for *L. decidua* and *F. sylvatica* where data were available for June, July and August (Appendix S6). The RWL at *P*_50_ was not related to wood density (r^2^ = 0.26, *P* = 0.16, n = 9). When excluding *P*. x *canescens*, which had high SWC (or low‐basic density) and a flat water release curve, a significant negative relationship between BD and the RWL at *P*_50_ could be established (r^2^ = 0.57, Fig. 3D). Since wood density decreased with increasing SWC, RWL at *P*_50_ increased with increasing SWC (r^2^ = 0.56, Fig. 3E). RWL at *P*_50_ was not significantly related to *P*_50_. When *P. abies* trunk wood was excluded from analysis (Fig. 3F), the relationship was still not significant, but we found a negative trend (r^2^ = 0.45, *P* = 0.07).

RWL and Ψ were not always linearly related in the species investigated (Fig. 1, Appendix S4). *P*_50_ calculated from direct PLC and modeled *P*_50_ from RWL at *P*_50_ and Ψ‐RWL curves showed a 1:1 relationship with an r^2^ of 0.99 (Fig. 2B). By means of the same equations and RWL at *P*_88_ (Appendix S4), it was as well possible to predict *P*_88_ (Table 1) with an r^2^ of 0.94 (*P* < 0.0001).

## Discussion

### Relative water loss curves: possible determinants for their shape

Relative water loss was a good predictor for the conductivity loss as indicated by the strong relationship between *P*_50_ calculated from hydraulic experiments and the empirically modeled *P*_50_ (Fig. 2B). The empirically modeled *P*_50_ was calculated from the RWL at *P*_50_ and RWL curves. RWL curves (Fig. 1) resembled those published for branch and stem segments of different conifer and angiosperm species (Domec and Gartner 2001, 2002, Domec et al. 2006, Gleason et al. 2014, Blackman et al. 2016). RWL curves with a steeper increase at high‐water potentials were linear (Fig. 1), as assumed by Gleason et al. (2014) and Blackman et al. (2016) for angiosperm branches of several species and as found by Domec and Gartner (2001) for conifer trunk wood. Especially in conifer branches and young stems (Fig. 1), the slopes of the RWL curves had a much less steep increase at the initial stage (at high Ψ) than those found in water release curves where Ψ was determined on small sapwood specimens by thermocouple psychrometry (Meinzer et al. 2003, Barnard et al. 2011, McCulloh et al. 2014, Jupa et al. 2016, Pratt and Jacobsen 2017) or in vacuum degassed and centrifuged 27 cm long samples (Pivovaroff et al. 2016). The difference in the shape of the water release curves obtained with the psychrometry method might be explained by differences in sample size. Very small samples have a high amount of cut open surfaces in relation to volume. Dehydration in such samples should be thus quite fast and proceeds from the outside to the inside rather than from the more vulnerable to the less vulnerable conduits as it is expected in bigger sapwood samples or branches. In our specimens, phase one, i.e. the water release from open conduits, damaged and non‐functional conduits and intercellular spaces (Pratt and Jacobsen 2017), was much less pronounced. In species with low vulnerability to cavitation, Ψ decrease at the beginning of dehydration resulted in low RWL. It is suggested that the steep increase thereafter was phase two, defined as the RWL due to cavitation and release from elastic storage. Phase three, the post cavitation phase, i.e. water loss when wood lost all of its hydraulic conductivity (Pratt and Jacobsen 2017), is missing in the RWL‐Ψ curves presented in Fig. 1 because experiments were performed above fiber saturation. At fiber saturation, cell lumina no longer contain free water, but cell walls are fully saturated with water (Berry and Roderick 2005). For a given method, differences in shape of the RWL curves between conifer branches and trunk wood or angiosperm branches might be due to the presence of reaction wood. Whereas the presence of compression wood in conifer branches and young trunks has an influence on hydraulic vulnerability (Mayr and Cochard 2003), no such impact has been reported for tension wood in angiosperms so far (Gartner et al. 2003, Badel et al. 2015).

### The relationship between conductivity loss and relative water loss is species‐ and age‐specific

PLC was strongly related to RWL as found in an earlier study of Hietz et al. (2008) for *P. abies* branches. We found no methodical influence on the relationship between PLC and RWL (Appendix S5). In other words, regardless how the drought stress was induced (or how this stress was measured at ambient temperatures, excluding extremely destructive methods), a given water loss should result in its corresponding loss of hydraulic conductivity. How much water loss is necessary to result in 50% loss of conductivity (Table 1) is most likely linked to wood structure that is extremely variable across species regarding structure–function relationships (Lachenbruch and McCulloh 2014). The relative water loss resulting in 50% loss of conductivity was surprisingly low in some species, especially for *F. sylvatica* saplings, where only 10% RWL were sufficient. In contrast, for *P. abies* trunk wood, 25% RWL was necessary to reach 50% loss of conductivity. According to earlier and recent studies (Meinzer et al. 2003, Domec et al. 2006, McCulloh et al. 2014, Trifilò et al. 2015, Jupa et al. 2016, Pratt and Jacobsen 2017), *F. sylvatica* would fall into a low‐capacitance category and *P. abies* trunk wood into the highest capacitance category. It is supposed that high‐capacitance buffers further decline in water potential because water withdrawal from intact, water‐filled, conduits during embolism formation contributes to the transpiration stream (Tyree and Zimmermann 2002, Hölttä et al. 2009, Vergeynst et al. 2015, Salomón et al. 2017).

### Hydraulic vulnerability and water storage capacitance

Are species or plant organs that have a relatively low amount of overall water contributing to hydraulic conductivity (‘low capacitance’) more successful in refilling their sapwood after drought events than species or organs with a high‐water withdrawal at a given decrease in Ψ (‘high capacitance’)? This question is of specific relevance since many angiosperm species operate at water potentials below *P*_50_ (Choat et al. 2012), which means on one hand that these plants allow a substantial decrease in hydraulic conductivity, and on the other hand that they must be capable of refilling the emptied conduits (Salomón et al. 2017). Accordingly, diurnal changes in conductivity loss (or PLC recovery) are more pronounced in angiosperm species with higher *P*_50_ (Trifilò et al. 2015). For young stems and branches, we found a negative trend across species between *P*_50_ and RWL at *P*_50_ (Fig. 3F). This suggests that, in hydraulically more vulnerable species, removal of a lower relative amount from the stored water results in 50% conductivity loss. Regarding refilling processes in the juvenile woody parts of these species, a lower relative water amount should be thus necessary to regain full sapwood conductivity. To detect a negative trend between *P*_50_ and RWL at *P*_50_ we had to remove the dataset of the *P. abies* mature trunk wood samples. McCulloh et al. (2014) report that mature trunk wood of four conifer species had higher hydraulic vulnerability together with higher hydraulic capacitance than branches. For *P. abies*, where we investigated branches, saplings and sapwood of the main trunk, a similar trend was found (Fig. 3F). The main trunk of mature conifer trees may serve as a water storage reservoir indicated by its high SWC, whereas conifer branches and saplings have a much higher safety against cavitation combined with lower SWC and RWL at *P*_50_. Differences in hydraulic vulnerability within a stem (age‐specific) and between plant organs (roots, trunk, branches) have been reported for conifers (Domec and Gartner 2002, Domec et al. 2009, Rosner et al. 2014) and for angiosperms (Charrier et al. 2016). Interspecies‐ (Hacke et al. 2001a, Hacke and Jansen 2009, Lachenbruch and McCulloh 2014) or age‐specific variability of *P*_50_ in conifer stem axes is strongly related to wood density (Domec et al. 2009, Rosner 2017), but such relationships may be rather weak or do not necessarily exist for conifer branches (Martínez‐Vilalta et al. 2009, Corcuera et al. 2011, Lamy et al. 2012) or across angiosperm and conifer species (Gleason et al. 2016, Savi et al. 2017). Accordingly, we found no relationship between *P*_50_ and wood density across species.

High‐wood density can be associated with low capacitance (Pratt et al. 2007, Scholz et al. 2007, McCulloh et al. 2014, Trifilò et al. 2015, Pratt and Jacobsen 2017, Savi et al. 2017), as it was the case in *F. sylvatica* saplings and *S. torminalis* branches (Table 1). We found a trend for decreasing capacitance (expressed as lower relative water loss resulting in 50% of conductivity loss) with increasing wood density across species in accordance with the studies mentioned above. In contrast, *P*. x *canescens* saplings had high‐saturated water contents (or low‐basic density) in combination with quite flat water release curves, where only about 11% of RWL resulted in *P*_50_ and 20% in *P*_88_. In that regard, this species could be categorized as ‘extreme water saver’. When this species was excluded from analyses, a significant negative relationship between density and the RWL at *P*_50_ could be established (Fig. 3D). More studies on the RWL at 50 or 88% of conductivity loss are necessary in order to get a deeper knowledge on the different strategies plants have evolved to cope with drought. This could be especially interesting in the *P. tremula*/*P*. x *canescens*/*P. alba* hybrid system (e.g. Lexer et al. 2004), as it seems to combine, mix and possibly segregate (in later generations, e.g. Christe et al. 2016) different evolutionary ‘strategies’ for avoiding the effects of drought.

## Conclusions

The novel approach described in this study can be used for the prediction of the percent loss of conductivity by gravimetric measurements and for the detection of outliers that may come from erroneous flow measurements. The precondition for this new approach is the knowledge of the relationship between PLC and RWL that is not only species‐specific but may vary also across cambial age and with growth conditions. Once this relationship is established on a small parallel calibration sample set, hydraulic conductivity loss can be reliably, easily and fastly estimated by simple gravimetric measurements on a larger sample set. The quality of the empirical models can be validated by relating *P*_50_ calculated from RWL measurements to *P*_50_ from flow measurements across species. Potential applications are tree screenings, e.g. in genetic tests, for drought sensitivity and a fast interpretation of diurnal‐, seasonal‐ or drought‐induced changes in xylem water content upon their impact on hydraulic conductivity loss. It is thus suggested to quantify RWL in parallel with PLC when non‐automated, classical methods (bench top dehydration and air injection) are used, because the measurements take only some seconds but can be used to detect outliers and give information on a plant's survival strategies.

## Author contributions

B.H. provided plants for the poplar sample sets. The practical work was done by S.R. who was assisted by G.D.S. and T.S. in the period from 2016 to 2017. Data analysis was carried out by S.R., G.D.S. and T.S. contributed to the interpretation of the results. S.R. wrote a first draft of the manuscript; thereafter, B.H., G.D.S. and T.S. revised this draft by rewriting, discussion and commenting. All authors agree on the contents of this manuscript.

## Supporting information


**Appendix S1.** Information on the plant material
**Appendix S2.** Information on the hydraulic vulnerability curves
**Appendix S3.** Relationship between hydraulic conductivity loss and relative water loss
**Appendix S4.** Water loss curves and predicted water potential at 50 and 88% conductivity loss
**Appendix S5.** Loss of hydraulic conductivity and relative water loss assessed with different methods
**Appendix S6.** Loss of hydraulic conductivity and relative water loss and in different months
