# Supplementary material for: Prediction of hydraulic conductivity loss from relative water loss: new insights into water storage of tree stems and branches
Source: Physiol Plant. 2018 Sep 4;165(4):843–54. doi: 10.1111/ppl.12790 (PMC7379737; doi:10.1111/ppl.12790)
Supplement: Supplementary file 1 — Appendix S1. Information on the plant material Appendix S2. Information on the hydraulic vulnerability curves Appendix S3. Relationship between hydraulic conductivity loss and relative water loss Appendix S4. Water loss curves and predicted water potential at 50 and 88% conductivity loss Appendix S5. Loss of hydraulic conductivity and relative water loss assessed with different methods Appendix S6. Loss of hydraulic conductivity and relative water loss and in different months [file PPL-165-843-s001.pdf]

Supporting information

Appendix S1. Information on the plant material

Appendix S2. Information on the hydraulic vulnerability curves

Appendix S3. Relationship between hydraulic conductivity loss and relative water loss

Appendix S4. Water loss curves and predicted water potential at 50% and 88% conductivity loss

Appendix S5. Loss of hydraulic conductivity and relative water loss assessed with different methods

Appendix S6. Loss of hydraulic conductivity and relative water loss in different months

Appendix S1: Information on the origin of the plant material, on the number of tree individuals, the sample number as well as the dataset number (i.e. single hydraulic measurements). The numbers in brackets give information on the sample numbers measured with the bench top dehydration method.

**Species**	**Origin**	**Lat. N**	**Long E.**	**Altitude [m]**	**Tree age**	**Trees n**	**Organ**	**Samples n**	**Datasets n**
Picea abies	Botanical garden, Vienna, Austria	48°14′12″	16°20′15″	233	4	8	trunk sapling	8	54
Picea abies	Botanical garden, Vienna, Austria	48°14′12″	16°20′15″	233	20	5	branch	6	46
Picea abies	Sande, Southern Norway	59°34′50″	10°16′29″	105	40	6	trunk wood	18	44
Larix decidua	Botanical garden, Vienna, Austria	48°14′12″	16°20′15″	233	20	2	branch	8	52
Acer campestre	Botanical garden, Vienna, Austria	48°14′12″	16°20′15″	233	3-5	13 (7)	trunk sapling	16 (10)	59
Fagus sylvatica	Botanical garden, Vienna, Austria	48°14′12″	16°20′15″	233	3-6	37 (31)	trunk sapling	88 (82)	112
Populus x *canescens*	River “Wien”, Vienna, Austria	48°12'30"	16°13'34"	224	3-4	18	trunk sapling	27	101
Populus tremula	River “Wien”, Vienna, Austria	48°12'30"	16°13'34"	224	30	17 (13)	branch	45 (41)	65
Sorbus torminalis	Botanical garden, Vienna, Austria	48°14′12″	16°20′15″	233	60	1	branch	8	47

Appendix S2: Information on the hydraulic vulnerability curves of seven different woody species fitted by an exponential sigmoidal equation (percent loss of conductivity = 100/(1 + exp(*a**(pressure-*b*)))) (Pammenter and VanderWilligen 1998). The parameter "*a*" corresponds to the slope of the linear part of the regression and "*b*" is the P_{50}, i.e. the pressure that is necessary to result in 50% conductivity loss. P_{88} is the pressure that would result in 88% conductivity loss. Results for *a* and P_{50} are given with ± standard error and the 95% confidence interval (CI 95%) is given in brackets. Information on the sample- and dataset numbers is given in Table S1.

**Species**	***a***	**P_{50} [MPa]**	**r^2**	***P***
Picea abies sapling	0.74 ± 0.05	-6.31 ± 0.08 (-6.46 -6.15)	0.93	< 0.0001
Picea abies branch	1.62 ± 0.15	-4.63 ± 0.06 (-4.74 -4.51)	0.95	< 0.0001
Picea abies trunk wood	2.86 ± 0.27	-2.21 ± 0.03 (-2.27 -2.15)	0.94	< 0.0001
Larix decidua branch	1.11 ± 0.06	-4.45 ± 0.05 (-4.55 -4.34)	0.97	< 0.0001
Acer campestre sapling	0.82 ± 0.81	-4.17 ± 0.11 (-4.44 -3.98)	0.90	< 0.0001
Fagus sylvatica sapling	0.92 ± 0.06	-1.90 ± 0.05 (-2.00 -1.80)	0.83	< 0.0001
Populus x *canescens* sapling	2.01 ± 0.11	-3.17 ± 0.03 (-3.22 -3.11)	0.94	< 0.0001
Populus tremula branch	1.34 ± 0.13	-2.27 ± 0.07 (-2.41 -2.13)	0.82	< 0.0001
Sorbus torminalis branch	1.02 ± 0.08	-2.88 ± 0.07 (-3.02 -2.73)	0.93	< 0.0001

Appendix S3:: Parameters for linear (*b*0, *b*1), quadratic (*b*0, *b*1, *b*2) or cubic (*b*0, *b*1, *b*2, *b*3) equations for the relationship between percent loss of conductivity (PLC, independent variable) and the relative water loss (RWL, dependent variable) ($P < 0.0001$), the calculated RWL resulting in 50% (RWL at P_{50}) and 88% (RWL at P_{88}) of conductivity loss and their 95% confidence intervals (CI 95%) and 95% individual prediction intervals (PI 95%) for seven different temperate woody species and different age classes. Mean values are given with their standard error.

Species and organ	*b*0	*b*1	*b*2	*b*3	r^2	*P*	RWL at P_{50} (CI 95%, PI 95%) [%]	RWL at P_{88} (CI 95%, PI 95%) [%]
Picea abies sapling	0.9642	0.4001			0.94	< 0.0001	20.97 (19.98 21.96, 15.41 26.54)	36.17 (34.26 38.09, 30.37 41.97)
Picea abies branch	1.7912	0.3703	-0.00147	3.028E-5	0.98	< 0.0001	20.41 (19.60 21.21, 15.07 25.74)	43.60 (42.30 44.89, 38.31 48.88)
Picea abies trunk wood	-0.7449	0.5076			0.96	< 0.0001	24.64 (23.55 25.72, 17.40 31.87)	43.93 (42.27 45.59, 36.58 51.27)
Larix decidua branch	1.3540	0.0617	0.00426		0.98	< 0.0001	15.09 (14.46 15.71, 10.61 19.57)	39.77 (38.75 40.79, 35.28 44.26)
Acer campestre sapling	0.2635	0.3866			0.89	< 0.0001	19.59 (18.27 20.91, 10.34 28.84)	34.28 (32.16 36.40, 24.89 43.68)
Fagus sylvatica sapling	-0.6300	0.2139			0.85	< 0.0001	10.07 (9.65 10.48, 5.59 14.54)	18.19 (17.43 18.96, 13.68 22.71)
Populus x *canescens* sapling	0.1207	0.3731	-0.00470	3.482E-5	0.94	< 0.0001	11.38 (10.95 11.81, 7.48 15.29)	20.29 (19.55 21.04, 16.45 24.14)
Populus tremula branch	-2.1077	0.4203			0.90	< 0.0001	18.90 (17.81 20.00, 10.11 27.70)	34.87 (33.01 36.74, 25.95 43.80)
Sorbus torminalis branch	1.3535	0.2462			0.91	< 0.0001	13.66 (12.91 14.41, 8.62 18.71)	23.02 (21.75-24.29, 17.87-28.17)

Appendix S4: Relationship between pressure application (air injection method) or water potential (bench top dehydration method) and the relative water loss (RWL, independent variable) for the prediction of P_{50}. Equation parameters for the pooled dataset are given either for linear (*b*0, *b*1), quadratic (*b*0, *b*1, *b*2) or cubic (*b*0, *b*1, *b*2, *b*3) equations for branches, saplings or trunk wood of seven different temperate woody species. The predicted values of P_{50} and P_{88} are given with their 95% confidence intervals (95% CI) and 95% prediction intervals (95% PI). The point measurements for the equations are shown in Fig. 1, the RWL at 50% or 88% of conductivity in Table 1.

**Species/organ**	***b*0**	***b*1**	***b*2**	***b*3**	r^2	***P***	**P_{50} pred. (95% CI, 95% PI)** [MPa]	**P_{88} pred. (95% CI, 95% PI)** [MPa]
Picea abies sapling	-0.4061	-0.6779	0.0266	-0.0004	0.95	< 0.0001	-6.43 (-6.63 -6.22, -7.61 -5.24)	-8.15 (-8.54 -7.76, -9.54 -6.76)
Picea abies branch	-1.2307	-0.2109	0.0021		0.87	< 0.0001	-4.65 (-4.89 -4.40, -6.35 -2.95)	-6.39 (-6.83 -5.98, -8.09 -4.70)
Picea abies trunk wood	-0.9150	-0.0505			0.83	< 0.0001	-2.16 (-2.29 -2.03, -3.02 -1.30)	-3.13 (-3.33 -2.94, -4.00 -2.26)
Larix decidua branch	-1.0639	-0.2530	0.0028		0.91	< 0.0001	-4.24 (-4.45 -4.04, -5.74 -2.74)	-6.68 (-7.05 -6.31, -8.19 -5.17)
Acer campestre sapling	-1.5801	-0.1308			0.78	< 0.0001	-4.14 (-4.42 -3.86, -5.99 -2.30)	-6.06 (-6.48 -5.65, -7.93 -4.20)
Fagus sylvatica sapling	-0.2166	-0.1424	-0.0025		0.84	< 0.0001	-1.90 (-1.99 -1.81, -2.88 -0.92)	-3.62 (-3.78 -3.46, -4.61 -2.63)
Populus x *canescens* sapling	-0.2088	-0.4928	0.0247	-0.0004	0.92	< 0.0001	-3.28 (-3.37 -3.19, -4.14 -2.43)	-3.81 (-3.97 -3.65, -4.68 -2.95)
Populus tremula branch	-0.6723	-0.0874			0.76	< 0.0001	-2.32 (-2.49 -2.15, -3.68 -0.96)	-3.72 (-4.00 -3.44, -5.10 -2.34)
Sorbus torminalis branch	-0.1901	-0.1892			0.91	< 0.0001	-2.78 (-2.92 -2.63, -3.77 -1.78)	-4.54 (-4.79 -4.30, -5.55 -3.53)

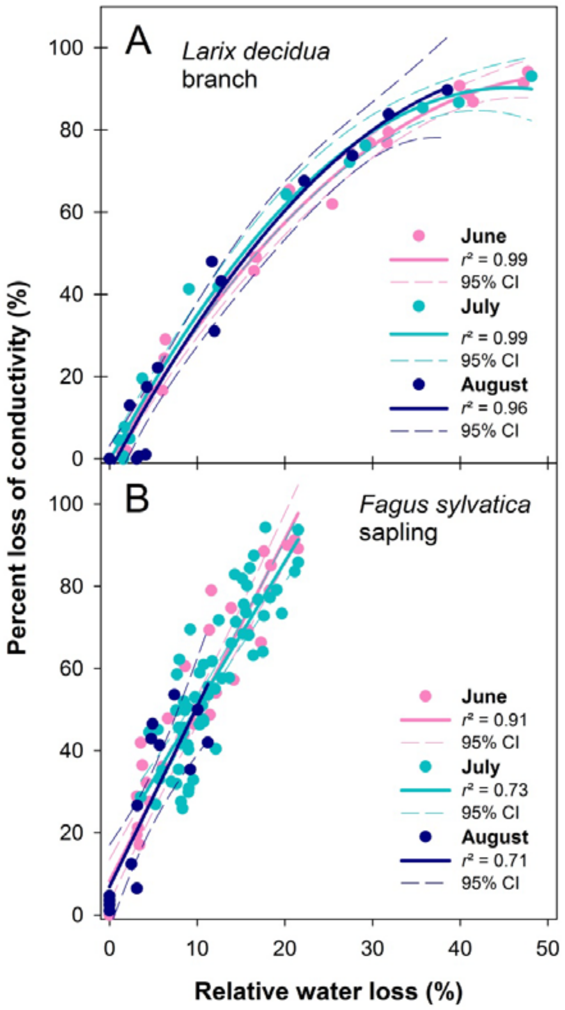

Appendix S5: Relationship between loss of hydraulic conductivity and relative water loss assessed with the bench top dehydration method and the air injection method for *Acer campestre* saplings (A), *Fagus sylvatica* saplings (B) and *Populus tremula* branches (C). The linear equations pooled for different hydraulic methods have overlapping 95% confidence intervals (95% CI).

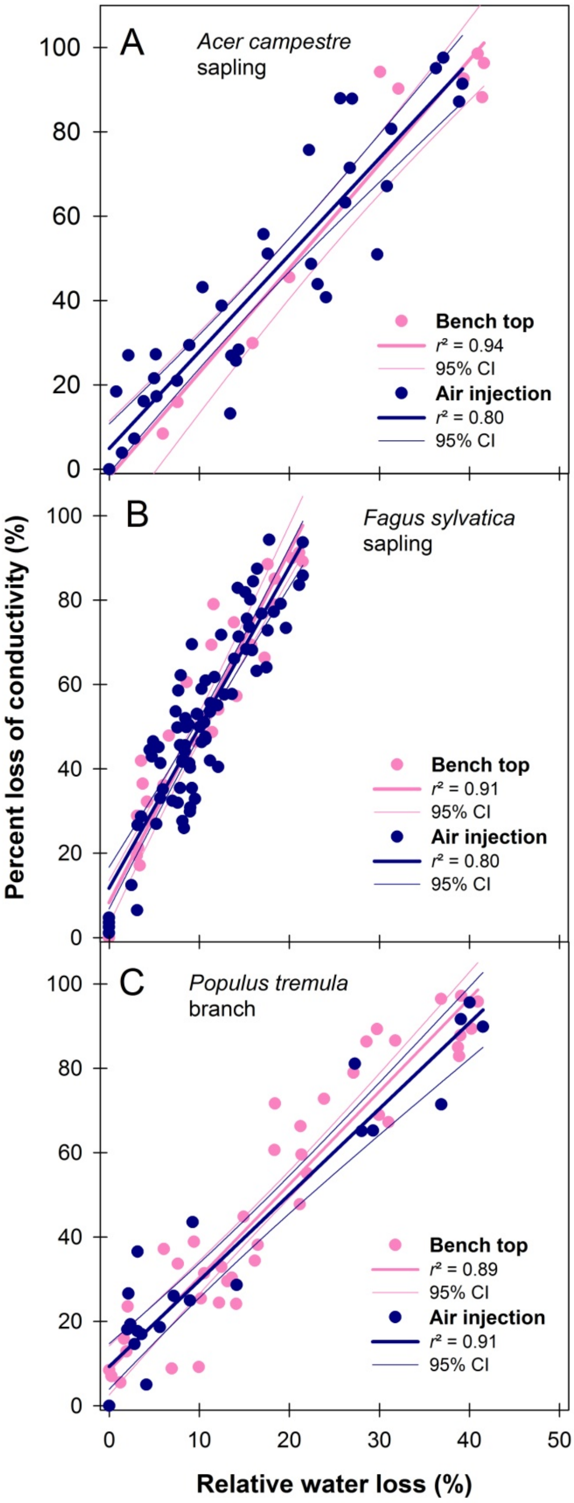

Appendix S6: Relationship between loss of hydraulic conductivity and relative water loss in different months (June, July and August) for *Larix decidua* branches (A) and *Fagus sylvatica* saplings (B). The quadratic (A) and linear (B) equations pooled for different months have overlapping 95% confidence intervals (95% CI).
